# *M. tuberculosis* Infection Attributable to Exposure in Social Networks of Tuberculosis Cases in an Urban African Community

**DOI:** 10.1093/ofid/ofae200

**Published:** 2024-04-16

**Authors:** Noah Kiwanuka, Sarah Zalwango, Robert Kakaire, Maria Eugenia Castellanos, Trang Ho Thu Quach, Christopher C Whalen

**Affiliations:** Department of Epidemiology and Biostatistics, School of Public Health, College of Health Sciences, Makerere University, Kampala, Uganda; Department of Public Health and Environment, Kampala Capital City Authority, Kampala, Uganda; Global Health Institute, College of Public Health, University of Georgia, Athens, Georgia, USA; Public Health and Tropical Medicine, College of Public Health, Medical and Veterinary Sciences, James Cook University, Townsville, Australia; Global Health Institute, College of Public Health, University of Georgia, Athens, Georgia, USA; Global Health Institute, College of Public Health, University of Georgia, Athens, Georgia, USA

**Keywords:** latent tuberculosis infection, population-attributable fraction, tuberculosis, tuberculosis control

## Abstract

**Background:**

The persistence of tuberculosis today and its global disparity send a powerful message that effective tuberculosis control must respond to its regional epidemiology. Active case finding through contact investigation is a standard protocol used for tuberculosis control, but its effectiveness has not been established, especially in endemic areas.

**Methods:**

To quantify the potential effectiveness of contact investigation in Kampala, Uganda, we used a cross-sectional design to evaluate the social networks of 123 tuberculosis index cases and 124 controls without tuberculosis.

**Results:**

Tuberculous infection was present in 515 of 989 tuberculosis case contacts (52.1%) and 396 of 1026 control contacts (38.6%; adjusted prevalence ratio, 1.4; 95% CI, 1.3–1.6). The proportion of infected participants with known exposure within the social network of the tuberculosis case was 35%. The population-attributable fraction was 11.1% for any known exposure, with 7.3% attributable to household exposure and 3.4% attributable to extrahousehold exposure.

**Conclusions:**

This low population-attributable fraction indicates that contact tracing in the social networks of index cases will have only a modest effect in reducing tuberculous infection in a community. New approaches to community-level active case finding are needed.

Infection with *Mycobacterium tuberculosis* persists in many parts of the world today, especially Sub-Saharan Africa. Tuberculosis is one of the leading causes of death worldwide and the foremost cause of death from an infectious disease [1]. Tuberculosis persists in regions because 1 case is replaced by at least 1 other case emerging from the pool of individuals with latent or newly acquired infection [2, 3]. In these regions, national tuberculosis control programs are unable to curtail this replacement of tuberculosis cases, so the disease remains endemic. The global disparity in tuberculosis disease sends a powerful message that effective tuberculosis control must respond to regional epidemiology and address local determinants of epidemic behavior.

For decades, tuberculosis contact investigation has been used as a mainstay of active case finding, especially in the households of tuberculosis cases [4]. The objective of contact investigation is to identify, report, and treat undetected cases of tuberculosis among contacts of an index case and to screen contacts without disease for latent tuberculous infection [5]. The household of an index case is a natural choice for contact investigation because the household is a setting of intense transmission of *M. tuberculosis* to household members [6, 7]. Active case finding in this setting is important for tuberculosis care because it may reduce morbidity and mortality of tuberculosis among vulnerable contacts [8, 9] and create opportunities to provide tuberculosis preventive therapy [10].

Whether contact investigation of the household alone is an effective intervention in controlling tuberculosis at the community level is another question altogether and remains unanswered [11–14]. For years, it was thought that most transmission in a community occurred in the households of tuberculosis index cases [4, 15–17], until a series of studies began to question this premise. Now, it appears that household exposure to an index case accounts for <14% of infection in a community [18–20]. A natural extension of household contact investigation is to evaluate contacts outside the household who may be at high risk of tuberculosis or latent tuberculosis infection (LTBI), such as close friends, relatives, or work associates. Despite the common sense of this approach, its incremental effectiveness in contact tracing is unknown because evaluation of contacts beyond the household becomes more challenging to implement in low- and middle-income countries [21–23].

As control of tuberculosis depends on reducing transmission or preventing progression of infection to disease, we framed our research question to ask whether contact investigation in the social network of an index case reduces tuberculous infection—both latent tuberculosis infection and disease—in a community with endemic tuberculosis. To address this question, we expanded our previous analysis of the excess risk of household and social network exposure [24] to calculate the population-attributable fraction (PAF) [25], which estimates the proportion of tuberculous infection in the community that may be attributable to social network exposure. This study builds a valid framework for estimating the effectiveness [26] of contact investigation in limiting infection in the community and for informing policy for tuberculosis control.

## METHODS

This was a cross-sectional study conducted in Kampala, Uganda, in which we enrolled a consecutive sample of 123 index cases of pulmonary tuberculosis who presented to the Ugandan National Tuberculosis and Leprosy Programme between July 2013 and February 2017 [24]. All cases were age 15 years or older, residents of Lubaga Division of Kampala, and had a confirmed microbiological diagnosis using sputum microscopy, Xpert MTB/RIF (Cepheid, Sunnyvale, CA, USA), or culture. Extrapulmonary tuberculosis and pediatric tuberculosis were excluded because of the minimal risk of transmission. As a comparison group, we enrolled 124 adult residents of Lubaga Division without tuberculosis at the time of enrollment in the study (Supplementary Data). As none of the selected controls were diagnosed with tuberculosis during the study period, we defined them as index controls. Index tuberculosis cases and index controls were frequency-matched by age category, sex, and parish of residence in Lubaga. The sample size was determined based on the risk difference in tuberculous infection of 5% between contacts of index cases and community controls, 80% power, and 5% error.

The social networks of index cases and index controls were ascertained using the same procedures. To identify the members of the social networks of index participants, we performed structured interviews with each index participant and asked the participant to list individuals with whom they had a personal relationship [27]. We began by listing household members and family members; we next listed other groups of contacts who were considered close as described by the participant. To enhance recall, we used recent time frames and sociological prompts familiar to Ugandans, such as work, recreation or free time, family or social responsibilities, and hobbies [28–31]. Contacts were classified as household contacts if they resided in the household of an index participant for the previous 3 months and had eaten meals in the household at least weekly [24]; otherwise, contacts were classified as extrahousehold.

We defined exposure as recent or past membership in the social network of a tuberculosis case. Recent exposure was classified as reported exposure to a tuberculosis case within the preceding year before enrollment in the study. All contacts of index cases were classified as recent; control contacts were classified as recent if they reported contact with a tuberculosis case within a year. Cumulative exposure to tuberculosis was defined as remote contact with a tuberculosis case at any time in the past and was ascertained through self-report from all contacts, both of index cases and index controls.

Trained interviewers collected demographic and social characteristics of the index participants and their contacts. Index participants and their contacts in the study were evaluated for tuberculous infection. Tuberculous infection was defined as infection with *M. tuberculosis* and was classified as either LTBI or tuberculosis disease. LTBI was defined as a reactive tuberculin skin test (TST) [24] in a participant without signs or symptoms of disease. A reactive TST was defined as an induration of 10 mm or greater among HIV-seronegative contacts and 5 mm among HIV-seropositive contacts [5, 32]. Tuberculosis disease was defined in a participant who had signs or symptoms of disease and confirmed microbiological diagnosis using sputum microscopy, Xpert MTB/RIF (Cepheid, USA), or mycobacterial culture. Chest x-rays were not taken on asymptomatic contacts because of perceived risk of radiation. Contacts who did not meet the definitions for tuberculous infection were classified as uninfected.

### Statistical Analyses

The age–sex prevalence of tuberculous infection was estimated for all index contacts. Crude and adjusted prevalence ratios (PRs) with 95% CIs were estimated using a Poisson regression model with robust variance [33] to compare infection among social network members of index cases and index controls. The final regression models included age and sex of the contacts and sex and HIV status of the index participants. Bacillus Calmette-Guérin (BCG), HIV status of contact, and age of index were excluded from the final model because they did not confound the model nor improve its fit. Age of the contact was found to interact with the exposure variable; it was categorized as a binary variable to facilitate the interpretation of interaction terms.

The PAF for tuberculous infection represents the proportional reduction in tuberculous infection in a population that would occur if known exposure to an index case through their social network were mitigated in that population [34]. We estimated the PAF for tuberculous infection with a standard formula that used the PR of infection among contacts of cases vs controls and the probability of exposure given tuberculous infection [20, 26, 35]. To estimate the probability of social network exposure to infectious tuberculosis (both household and extrahousehold), we built a demographic model to create a standard population (Supplementary material) using prevalence of tuberculosis in Lubaga Division [36], demographic information of participants, observed age–sex prevalence of tuberculous infection, and self-reported contact with a tuberculosis case. Using this standardized population, we calculated the probability of household and extrahousehold exposure to a case of tuberculosis among infected persons. This analysis was then stratified by age category and sex separately. To assess uncertainty in the measurement of known prior exposure on the PAF, we performed a sensitivity analysis by varying the probability of exposure given infection from 0 to 1 and the PR from 1 to 10. Analyses were done using SAS software, version 9.4 (SAS Institute, Cary, NC, USA), and Stata 14.2 (StataCorp, College Station, Texas, USA).

### Patient Consent

The study was conducted in accordance with the ethical procedures of the Helsinki Declaration [37]. Adult participants provided written informed consent; minor participants age 15–17 years provided written informed assent, with written consent provided by a parent or legal adult guardian. Information was anonymized by using identification numbers instead of names. The University of Georgia Institutional Review Board, the Higher Degrees Research and Ethics Committee at Makerere University School of Public Health, and the Uganda National Council for Science and Technology all approved the study.

## RESULTS

We enrolled 989 contacts of tuberculosis cases; of these, 380 (38%) were household contacts and 609 (62%) were extrahousehold contacts (Table 1). We also enrolled 1026 contacts of community controls without tuberculosis. Tuberculosis case contacts were younger, more likely to be female, and had a lower monthly income; both groups were similar with regards to HIV infection, BCG vaccination status, educational level, and marital status. The age distribution differed between household and extrahousehold contacts of tuberculosis cases; 47% of household contacts were under 15 years of age compared with 14% of extrahousehold contacts.

**Table 1.** Demographic and Social Characteristics of Social Network Contacts of Tuberculosis Index Cases and Index Controls From Kampala, Uganda

	Contacts of Index Tuberculosis Cases						Contacts of Index Controls	
	All Contacts n = 989		HH Contacts n = 380		EHH Contacts n = 609		n = 1026	
Characteristic	No.	%	No.	%	No.	%	No.	%
Gender								
Male	477	48	166	44	311	51	571	56
Female	512	52	214	56	298	49	455	44
Age, median [IQR], y	23 [13–31]		16 [6–26]		26 [20–32]		25 [19–31]	
Age, mean (SD), y	23 (14)		19 (15)		26 (12)		25 (11)	
Age (category)								
0–4 y	115	12	80	21	35	6	56	5
5–14 y	144	14	98	26	46	8	109	11
≥15 y	730	74	202	53	528	87	861	84
HIV serostatus								
Positive	81	8	25	7	56	9	64	6
Negative^a^	908	92	355	93	553	91	962	94
BCG vaccine								
Yes	823	83	329	86	494	81	855	83
No	83	8	31	8	52	8	100	10
Unknown/missing	83	8	20	5	63	10	71	7
Religion								
Christian	719	73	260	68	459	75	797	78
Muslim	260	26	116	30	144	24	226	22
Other/unknown	10	1	4	1	6	1	3	0
Income								
<56 US dollars/mo	752	76	322	85	430	71	709	69
>56 US dollars/mo	229	23	55	14	174	28	311	30
Unknown	8	1	3	1	5	1	6	1
Education (highest attained)								
None^b^	162	16	97	26	67	11	119	12
Primary level	356	36	139	36	217	36	400	39
Post primary level	469	47	144	38	325	53	507	49
Marital status								
Never married	565	57	271	71	294	48	508	50
Married	321	32	80	21	241	40	420	41
Other^c^	103	10	29	8	74	12	98	10

Abbreviations: BCG, Bacillus Calmette-Guérin; EHH, extrahousehold; HH, household; IQR, interquartile range.

^a^Includes children who were too young to be tested and refusals.

^b^Includes 2 nonhousehold contacts and 1 control contact with unknown education level.

^c^Other = separated/divorced/widowed and unknown status.

Of the 989 tuberculosis case contacts, 515 had tuberculous infection, for an overall prevalence of 52.1% (Table 2); 496 had LTBI and 19 tuberculosis disease. The prevalence of tuberculous infection was higher in the household contacts (61.8%) compared with extrahousehold contacts (46%) of tuberculosis cases. Of the 1026 contacts of community controls, 396 had tuberculous infection, for a prevalence of 38.6%; 392 had LTBI, and 4 had tuberculosis disease. Household contacts of tuberculosis cases had a prevalence of tuberculous infection of 56.6% or higher, regardless of age, sex, or HIV serostatus. In contrast, the prevalence of tuberculous infection in the extrahousehold tuberculosis case contacts varied according to sex and age and was highest among male contacts age >15 years (57.5%) and lowest among female contacts age <15 years (8.3%).

**Table 2.** Prevalence of Tuberculous Infection in Household and Extrahousehold Contacts of Tuberculosis Cases and Their Controls

Characteristic	Contacts of Index TB Cases									Contacts of Community Controls		
	All Contacts			Only Household			Only Extrahousehold					
	TI	Total	Prevalence of Infection, %	TI	Total	Prevalence of Infection, %	TI	Total	Prevalence of Infection, %	TI	Total	Prevalence of Infection, %
Total	515	989	52.1	235	380	61.8	280	609	46.0	396	1026	38.6
Age												
0–4 y	49	115	42.6	41	80	51.2	8	35	22.9	1	56	1.8
5–14 y	63	144	43.8	62	98	63.3	1	46	2.2	13	109	11.9
≥15 y	403	730	55.2	132	202	65.3	271	528	51.3	382	861	44.4
Sex												
Male												
All	261	477	54.7	102	166	61.4	159	311	51.1	264	571	46.2
0–4 y	32	70	45.7	26	49	53.1	6	21	28.6	1	27	3.7
5–14 y	30	70	42.9	30	46	65.2	0	24	0.0	8	54	14.8
≥15 y	199	337	59.1	46	71	64.8	153	266	57.5	255	490	52.0
Female												
All	254	512	49.6	133	214	62.1	121	298	40.6	132	455	29.0
0–4 y	17	45	37.8	15	31	48.4	2	14	14.3	0	29	0.0
5–14 y	33	74	44.6	32	52	61.5	1	22	4.5	5	55	9.1
≥15 y	204	393	51.9	86	131	65.6	118	262	45.0	127	371	34.2
HIV serostatus												
Positive	45	81	55.6	17	25	68.0	28	56	50.0	23	64	35.9
Negative	470	908	51.8	218	137	61.4	252	553	45.6	373	962	38.8

Includes latent tuberculosis infection or tuberculosis disease.

Abbreviation: TI, tuberculous infection.

Of 384 community control contacts with tuberculous infection, 60 contacts reported recent exposure to an index case: 31 reported household exposure, 26 reported extrahousehold exposure, and in 3 the exposure setting was unknown. Assuming household and extrahousehold contacts within the networks of index cases were exposed, the overall probability of recent exposure within the social network of a tuberculosis case was 23% in the standardized population (Table 3), with a probability of exposure of 11% for both household and extrahousehold contact. When using recent or remote exposure, the overall probability of exposure to a tuberculosis case within their social network was 35%.

**Table 3.** Derivation of the Prevalence of Exposure to the Social Network of a Tuberculosis Case in a Standardized Population (Supplementary Data) Using Self-reported Information From Infected Contacts of Cases and Community Controls About Recent or Remote Exposure

	Self-reported Exposure in Community Control Contacts		Standardized Community Controls		Self-reported Exposure in Index Case Contacts		Standardized Population (Sum of Standardized Community Control and Observed Case Contacts)	
Group	No. Infected	Exposure Among Infected, No. (%)	No. Infected	Exposure Among Infected, No. (%)	No. Infected	Exposure Among Infected, No. (%)	No. Infected	Exposure Among Infected, No. (%)
Recent exposure								
Overall population^a^	384	60 (16)	5552	868 (16)	515	515 (100)	6067	1383 (23)
Household	…	31 (8)	…	448 (8)	…	235 (46)	…	683 (11)
Extrahousehold	…	26 (7)	…	376 (7)	…	280 (54)	…	656 (11)
Unknown^c^	…	3 (1)	…	43 (1)	…	-	…	43 (1)
Recent or past exposure								
Overall population^b^	350	103 (29)	5552	1634 (29)	515	515 (100)	6067	2149 (35)
Household	…	44 (13)	…	698 (13)	…	235 (46)	…	933 (15)
Extrahousehold	…	59 (17)	…	936 (17)	…	280 (54)	…	1216 (20)

^a^Of the 396 infected community control network members, 12 participants were uncertain about exposure to a tuberculosis case network within 1 year of the interview.

^b^Of the 396 infected community control network members, 46 participants did not respond to the question about ever knowing a tuberculosis case.

^c^Three participants indicated exposure to an index case social network but were not able to classify it as a household or extrahousehold contact.

Contacts of tuberculosis cases had a higher adjusted risk of tuberculous infection compared with community controls, whether the exposure was classified as recent or remote (PR*_adj_*, 1.4) (Table 4). There was effect modification of the PR by site of contact as the PR differed between household and extrahousehold contacts (PR*_adj_*, 1.9 vs 1.2, respectively).

**Table 4.** Probability of Exposure Given Infection, Adjusted Prevalence Ratio, and Population-Attributable Fraction for Tuberculous Infection Resulting From Either Recent or Remote Infection, According to Household Membership, Age Category, and Sex

Contact Characteristics	*P* (Exposure| Infection)	Adjusted Prevalence Ratio^a^ (95% CI)	Population-Attributable Fraction (95% CI)
All contacts	.35	1.4 (1.3–1.6)	11.1 (8.6–13.3)
Household	.15	1.9 (1.7–2.2)	7.3 (6.4–8.2)
Extrahousehold	.20	1.2 (1.1–1.4)	3.4 (1.4–5.2)
<15 y	.03	1.9 (1.6–2.3)	1.4 (1.1–1.7)
≥15 y	.32	1.4 (1.2–1.5)	9.1 (5.3–10.7)
Male	.20	1.5 (1.4–1.7)	6.7 (5.7–8.2)
Female	.15	1.4 (1.2–1.5)	4.3 (2.5–5.0)

^a^Adjusted prevalence ratio by continuous age (within exposure category), by sex of contact, and by sex and HIV status of index case.

The PAF given recent or remote exposure was 11.1%, with 7.3% attributable to household exposure and 3.4% attributable to extrahousehold exposure (Table 4). When using the upper value of the 95% CI for observed values of infection and exposure, the PAF was 14.7%, indicating an upper limit of the PAF given the observed data. In an analysis stratified by age, the PAF for recent or remote exposure among children age <15 years was 1.4% (95% CI, 1.1%–1.7%), and for individuals age 15 years or older it was 9.1% (95% CI, 5.3%–10.7%). For women, the PAF was 4.3% (95% CI, 2.5%–5.0%), and for men it was 6.7% (95% CI, 5.7%–8.2%).

**Figure 1.**
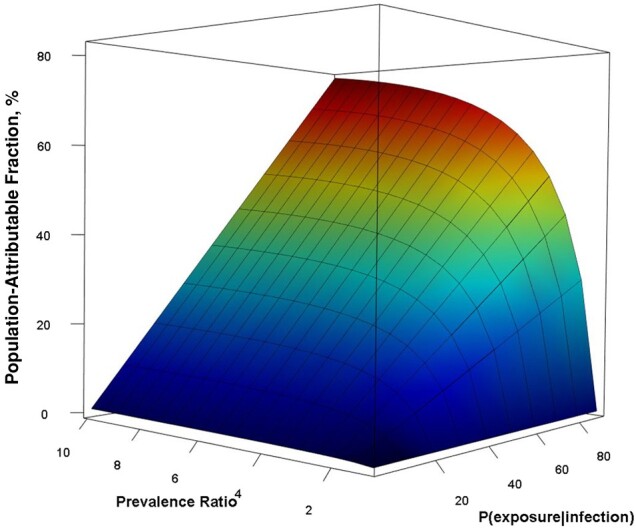
Sensitivity analysis population-attributable fraction according to probability of exposure given infection and the prevalence ratio. To generate the surface, we varied the probability of exposure given infection from 0 to 1 and prevalence ratio from 1 to 10 and calculated the population-attributable fraction for all possibilities.

The observed estimates of the PAR mapped to the lower portion of the surface calculated in the sensitivity analysis regardless of the definition of exposure proportion or use of upper limits of the PR (Figure 1). For reference, the minimum value of the PAF was 0 when either the PR equaled 1 or the probability of exposure was 0; the maximum value was 90% when the PR was 10 and the exposure proportion among infected individuals was 1.0. For any given exposure probability, the PAF was sensitive to change when the PR was low (ie, between 1 and 3) as it rose logarithmically but increased more gradually as the PR rose above 3. For any given PR, the PAF rose in a linear manner as the reported exposure proportion increased from 0 to 1.

## DISCUSSION

In an African city with endemic tuberculosis, we used the social networks of index tuberculosis cases and community controls to estimate the PAF of tuberculous infection given known exposure to tuberculosis within the social networks of index cases. We found that the PAF for known exposure was 11.1%. These findings indicate that if the effects of known exposure to infectious tuberculosis cases could be eliminated through interventions within these networks, such as contact investigation, the prevalence of tuberculous infection would decrease by only 11% with time. We also found that infection in the community was attributable to greater network exposure in contacts age >15 years, especially men.

**Figure 2.**
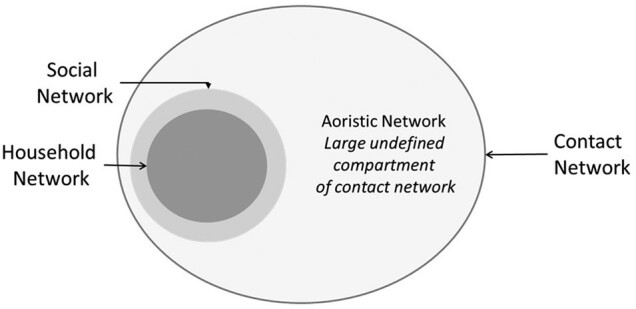
Schematic representation of the index tuberculosis case contact network, including household and extrahousehold social networks and indeterminate aoristic network.

We infer from these findings that the contact networks of tuberculosis cases are larger than their social networks (Figure 2). The contact network of an infectious index case comprises their social network and an aoristic network. The social network includes household and extrahousehold members who can be named by the case; the aoristic network includes casual or incidental contacts, most of whom are not named or known by the index case. According to our results, 89% of tuberculous infection in Lubaga Division of Kampala is attributed to exposure and transmission within this aoristic compartment of the index case contact network. Active case finding through household or social network contact investigation would not evaluate this large and influential portion of the contact network where most transmission occurs.

Our findings affirm previous molecular epidemiology studies of *M. tuberculosis* transmission that concluded that most transmission occurred outside the households or close contacts of index cases [18, 19]. We expand, however, on molecular studies in 2 important ways. First, our results are based on tuberculous infection and not on disease alone. Molecular studies rely on culture-confirmed tuberculosis cases and do not account for the latent period of tuberculous infection or the likelihood of progressing from asymptomatic infection to disease. Second, we present novel findings that show that extrahousehold contacts within the social networks of index cases account for only a marginal increment in the prevalence of infection in the community (3.4%).

The incomplete ascertainment of tuberculosis case contact networks underlies an inherent limitation of all contact investigation studies [38]. Here, it is important to distinguish between the social network—a list of known contacts of a case—and the wider contact network. Since the ascertainment of contact network depends on the index case, it will likely be incomplete because the case may not list all casual or incidental contacts from the infectious period. Although some active case finding studies have shown effectiveness in case detection, yield in case notification, and reduced mortality [9], none have conclusively shown a reduction in the prevalence of tuberculosis or latent infection [12–14]. In our study, even though we optimized network enrollment to obtain the maximum estimate possible for the PAF, it was only 14.7%. The inability to delineate this complete contact network imposes a real limitation on the potential effectiveness of contact tracing for reducing tuberculosis in cities with endemic disease.

As the prevalence of infectious tuberculosis and patterns of reported exposure may vary by age and sex across communities within cities or countries, the PAF may also vary. To explore this variability, we performed a sensitivity analysis in a standard population that was generated using a weighted average of the observed demographics, age- and sex-specific latent infection, and known exposure reported by contacts of index cases and index controls. We found that the PAF remained <20% for a wide range of plausible and observable values of infection prevalence and exposure within known networks, including results that are like ours. This analysis also indicates that if subclinical or pauci-bacillary tuberculosis [39, 40] led to a lower likelihood of known exposure than was observed, this type of misclassification would decrease the PAF.

According to our findings, active case finding would have a greater impact on tuberculosis control if it were performed in the aoristic compartment of the network. Although the patterns of transmission in the household of an index case are well known [6, 7, 16], little is known about the patterns of transmission in the community [41]. If we can develop reliable metrics for community transmission, they may be useful in designing focused community-based interventions [42, 43]. We already know from tuberculosis outbreak investigations that certain settings, such as bars [44], hospitals [45], health clinics [46], and congregate living [47], among others, act as transmission niches in the community. But these anecdotal reports do not provide a general model for understanding transmission in the community that can be used to design interventions.

We propose a theoretical framework for studying tuberculosis transmission that combines information from 3 types of networks, including the relational social network, a spatial network of locations visited by infectious cases, and the microbial network of *M. tuberculosis*. The social network of contacts is created in the weeks and months before diagnosis as the infectious index case infects known contacts whom they can identify [48]. The spatial network is created during this infectious period as the index case moves about their neighborhood or city to locations where they meet their social contacts or interact with casual or incidental contacts. This movement traces a pattern of locations where transmission may occur and may be measured using self-report or archived cellular telephone data [49, 50]. Both the social and geographic networks are linked through the microbial network [51] of *M. tuberculosis* strains that represents the evolutionary relationships between the strains transmitted by infectious cases in the population.

In an endemic setting, these 3 networks of index cases may overlap, thereby generating a larger sociocentric network that links multiple cases, their contacts, and locations in a community. Using a Bayesian model, we can analyze information from the 3 types of networks to infer geographic transmission hubs. Information about geographic transmission may provide direction about where to conduct interventions in a community with endemic disease [52].

The PAF was formulated using the PR, which compares the prevalence of infection among the exposed and unexposed and the likelihood of exposure among infected persons [26]. The internal validity of our findings depends on how we measured infection and exposure within social networks. To measure infection, we used the TST with standard criteria for infection. Although the TST is the traditional test for assessing latent tuberculosis infection and has well-understood performance characteristics in Uganda [53–56], we may have overestimated the prevalence of tuberculous infection because of false-positive tests associated with BCG vaccination and environmental mycobacterial infection. We could have used an interferon-gamma release assay, which is more specific [57], but its performance characteristics are not well established in African settings [58]. Despite its limitations, we believe that the TST was a reasonable metric because the PR would be unbiased as long as misclassification was similar among exposed and unexposed individuals.

The second parameter was the proportion of infected individuals with known exposure to a case of tuberculosis. By definition, all infected persons have been exposed to an infectious tuberculosis case, but since contacts may not recall, or may be unaware of, their exposure(s), self-reported information about exposure will likely be incomplete. To accommodate for the incomplete recall, we used a broad definition of social network, ascertained the membership of social networks through standardized interviews, used memory prompts and defined time frames, and did not exclude unspecified exposures in the community beyond identified social networks. As illustrated in the sensitivity analysis, the recall of exposure did not greatly affect the PAF when the prevalence of tuberculous infection in the community was high. For instance, when the PR was around 2, the PAF remained <20%, even when the known exposure to a tuberculosis case approached 100%.

In this study, we found that exposure in the households or social networks of tuberculosis cases accounted for only 10%–15% of tuberculous infection in an urban African community with endemic disease. This observation implies that most transmission resulting in infection occurred beyond the reported social network of the index case in an aoristic compartment of the contact network. If this finding is valid, then active case finding through household or close contact investigation would have minimal effect in reducing the prevalence of tuberculous infection in the community. Case finding in the wider contact network, however, may have a greater effect on tuberculosis control if new and effective methods for community-based case detection could be developed and implemented [14, 42, 59]. We propose that new approaches to case finding in the wider contact network include mobility of tuberculosis cases during their infectious periods and the locations where they visited and spent time.

## Supplementary Material

ofae200_Supplementary_Data

## Supplementary Data

Supplementary materials are available at *Open Forum Infectious Diseases* online. Consisting of data provided by the authors to benefit the reader, the posted materials are not copyedited and are the sole responsibility of the authors, so questions or comments should be addressed to the corresponding author.
