# Supplementary material for: M. tuberculosis Infection Attributable to Exposure in Social Networks of Tuberculosis Cases in an Urban African Community
Source: Open Forum Infect Dis. 2024 Apr 16;11(5):ofae200. doi: 10.1093/ofid/ofae200 (PMC11083641; doi:10.1093/ofid/ofae200)
Supplement: ofae200_Supplementary_Data [file ofae200_supplementary_data.docx]

Supplemental Materials

**A. Demographic Model of Standard Population**

This appendix describes the steps taken to develop a standardized population in which to estimate the prevalence of exposure among infected – P(exposure|infection).

1. We calculated the size of source population. Based on data from our research group, we estimated 0.8% as the prevalence of tuberculosis disease in Kampala (Sekandi et al., 2014). Since we enrolled 123 tuberculosis cases, the size of the source population is 15,375 persons (i.e., 123/0.008). This represents the size of the population that gave rise to the observed cases given the current prevalence of infectious tuberculosis in the community.
2. We enrolled 989 case contacts, with a size and age-sex distribution as follows:

**S1 Table. Demographic characteristics of 989 case contacts.**

| Sex | Age (years) | Total | Proportion of total |
| --- | --- | --- | --- |
| Male | <15 | 140 | 0.14 |
|  | >15 | 337 | 0.34 |
| Female | <15 | 119 | 0.12 |
|  | >15 | 393 | 0.40 |
| TOTAL |  | 989 | 1.00 |

1. We also estimated the observed age-sex tuberculosis infection prevalence (TBI) from case contacts.

**S2 Table. Estimated observed age-sex prevalence of tuberculosis infection from case contacts**

|  |  | Observed case contacts | | |
| --- | --- | --- | --- | --- |
| Sex | Age (years) | Infected | Total | Prevalence TBI |
| Male | <15 | 62 | 140 | 0.44 |
|  | >15 | 199 | 337 | 0.59 |
| Female | <15 | 50 | 119 | 0.42 |
|  | >15 | 204 | 393 | 0.52 |
| TOTAL |  | 515 | 989 | 0.52 |

1. To obtain the size of the control source population, we subtracted out the case contacts from the overall source population to obtain 14,386 (i.e., 15,375 – 989)
2. We used the age and sex distributions of the 1026 control contacts who were enrolled in the study to obtain the same distribution in the standardized population of controls.

**S3 Table. Observed and standardized prevalence of tuberculosis infection stratified by age category and sex.**

|  |  | Observed community controls | | Standardized  Control source population | |
| --- | --- | --- | --- | --- | --- |
| Sex | Age (years) | Total | Proportion of total | Total | Proportion of total |
| Male | <15 | 81 | 0.08 | 1136 | 0.08 |
|  | >15 | 490 | 0.48 | 6870 | 0.48 |
| Female | <15 | 84 | 0.08 | 1178 | 0.08 |
|  | >15 | 371 | 0.36 | 5202 | 0.36 |
| TOTAL |  | 1026 | 1.00 | 14386 | 1.00 |

1. We used the observed age-sex tuberculosis infection prevalence (TBI) from 1026 control contacts to calculate the number of infections in the control source population.

**S4 Table. Estimated number of tuberculosis infections in the observed and standardized control population stratified by age category and sex.**

|  |  | Observed Community  Controls (N = 1026) | | | Standardized Control  Source Population (N = 14,386) | | |
| --- | --- | --- | --- | --- | --- | --- | --- |
| Sex | Age (yrs) | Infected | Total | Prevalence TBI | Infected | Total | Prevalence TBI |
| Male | <15 | 9 | 81 | 0.11 | 126 | 1136 | 0.11 |
|  | >15 | 255 | 490 | 0.52 | 3575 | 6870 | 0.52 |
| Female | <15 | 5 | 84 | 0.06 | 70 | 1178 | 0.06 |
|  | >15 | 127 | 371 | 0.34 | 1781 | 5202 | 0.34 |
| TOTAL |  | 396 | 1026 | 0.39 | 5552 | 14386 | 0.39 |

1. From the community controls, we used their self-reported information about contact with an infectious case network as a way to determine the number of infected community members who were exposed to a network of an infectious case.

**S5 Table. Self-reported exposure to tuberculosis network in community controls.**

|  | Self-Reported Exposure in community control contacts | |
| --- | --- | --- |
| Group | No. infected | P(exposure\|infection)  n (%) |
| Recent Exposure |  |  |
| Overall population† | 384 | 60 (16) |
| Household |  | 31 (8) |
| Extra-household |  | 26 (7) |
| Unknown ○ |  | 3 (1) |
|  |  |  |
| < 15 years |  | 0 (0) |
| ≥ 15 years |  | 60 (16) |
|  |  |  |
| Male |  | 35 (9) |
| Female |  | 25 (7) |
| Recent or Past Exposure |  |  |
| Overall population‡ | 350 | 103 (29) |
| Household |  | 44 (13) |
| Extra-household |  | 59 (17) |
|  |  |  |
| < 15 years |  | 3 (1) |
| ≥ 15 years |  | 100 (28) |
|  |  |  |
| Male |  | 62 (18) |
| Female |  | 41 (11) |

† Of the 396 infected community control network members, 12 participants were uncertain about exposure to a tuberculosis case network within one year of the interview.

‡ Of the 396 infected community control network members, 46 participants did not respond to the question about ever knowing a tuberculosis case

○ Three participants indicated exposure to an index case social network but were not able to classify as household or extra household contact.

1. We combined age-sex specific categories to generate a standardized population with known tuberculous infection and exposure to infectious case. In this standardized population we estimated the prevalence of exposure among infected in this standardized population.

**S6 Table. Standardized populations with tuberculosis infection stratified by category of age and sex**

|  | Standardized community controls | | | Self-Reported Exposure in index case contacts | | | Standardized population (sum of standardized community control and observed case contacts) | |
| --- | --- | --- | --- | --- | --- | --- | --- | --- |
| Group | No.  infected | P(exposure\|infection)  n (%) | No.  infected | | P(exposure\|infection)  n (%) | No.  infected | | P(exposure\|infection)  n (%) |
| Recent Exposure  Overall population | 5552 | 868 (16) | 515 | | 515 (100) | 6067 | | 1383 (23) |
| Household |  | 448 (8) |  | | 235 (46) |  | | 683 (11) |
| Extra-household |  | 376 (7) |  | | 280 (54) |  | | 656 (11) |
| Unknown |  | 43 (1) |  | | - |  | | 43 (1) |
|  |  |  |  | |  |  | |  |
| < 15 years |  | 0 (0) |  | | 112 (22) |  | | 112 (2) |
| ≥ 15 years |  | 868 (16) |  | | 403 (78) |  | | 1271 (21) |
|  |  |  |  | |  |  | |  |
| Male |  | 506 (9) |  | | 261 (51) |  | | 767 (13) |
| Female |  | 362 (7) |  | | 254 (49) |  | | 616 (10) |
| Recent or Past Exposure  Overall population | 5552 | 1634 (29) | 515 | | 515 (100) | 6067 | | 2149 (35) |
| Household |  | 698 (13) |  | | 235 (46) |  | | 933 (15) |
| Extra-household |  | 936 (17) |  | | 280 (54) |  | | 1216 (20) |
|  |  |  |  | |  |  | |  |
| < 15 years |  | 48 (1) |  | | 112 (22) |  | | 160 (3) |
| ≥ 15 years |  | 1586 (28) |  | | 403 (78) |  | | 1989 (32) |
|  |  |  |  | |  |  | |  |
| Male |  | 984 (18) |  | | 261 (51) |  | | 1245 (20) |
| Female |  | 650 (11) |  | | 254 (49) |  | | 904 (15) |

**B. Characteristics of index cases and index controls**

| S7 Table. Comparison of demographic and clinical characteristics of tuberculosis index cases and matched index controls | | | | |
| --- | --- | --- | --- | --- |
| Characteristics | TB Cases N=123 (%) | Matched Controls N=124 (%) | ALL N=247 | P-value |
| Sex |  |  |  |  |
| Male | 84 | 85 | 169 | 1.000 |
| Female | 39 | 39 | 78 |  |
| Age Category |  |  |  |  |
| 15-19 | 5 (4.1) | 3 (2.4) | 8 (3.2) | 0.392 |
| 20-29 | 59 (48.0) | 49 (39.5) | 108 (43.7) |  |
| 30-39 | 37 (30.1) | 42 (33.9) | 79 (32.0) |  |
| 40+ | 22 (17.9) | 30 (24.2) | 52 (21.1) |  |
| HIV Status |  |  |  | 0.002 |
| Positive | 20 (16.3) | 6 (4.8) | 26 (10.5) |  |
| Negative | 98 (79.7) | 118 (95.2) | 216 (87.4) |  |
| Other | 5 (4.1) | 0 (0.0) | 5 (2.0) |  |
| Marital status |  |  |  | <0.001 |
| Married | 57 (46.3) | 91 (73.4) | 148 (59.9) |  |
| Not married | 66 (53.7) | 33 (26.6) | 99 (40.1) |  |
| Religion |  |  |  | 0.785 |
| Christians | 86 (69.9) | 89 (71.8) | 175 (70.9) |  |
| Muslim | 27 (22.0) | 28 (22.6) | 55 (22.3) |  |
| Other | 10 (8.1) | 7 (5.6) | 17 (6.9) |  |
| Education |  |  |  | 0.170 |
| None | 7 (5.7) | 2 (1.6) | 9 (3.6) |  |
| Primary level | 41 (33.3) | 49 (39.5) | 90 (36.4) |  |
| Post primary level | 75 (61.0) | 73 (58.9) | 148 (59.9) |  |
| Monthly income |  |  |  | 0.089 |
| < $80 | 88 (71.5) | 75 (60.5) | 163 (66.0) |  |
| ≥ $80 | 35 (28.5) | 49 (39.5) | 84 (34.0) |  |
| Ego-centric network size (mean, IQR) | 8 (7 – 9) | 8 (7 – 9) | 8 (7 – 9) | 0.437 |
| Household | 3 (1 – 5) | 1 (0 – 3) | 2 (0 – 4) | <0.001 |
| Extra-household | 5 (3 – 7) | 7 (5 – 8) | 6 (4 – 8) | <0.001 |

TB - tuberculosis
